# Supplementary material for: MetaRibo-Seq measures translation in microbiomes
Source: Nat Commun. 2020 Jun 29;11:3268. doi: 10.1038/s41467-020-17081-z (PMC7324362; doi:10.1038/s41467-020-17081-z)
Supplement: Supplementary file 10 — Supplementary Data 7 [file 41467_2020_17081_MOESM10_ESM.zip › File2/Confidence_High_Taxonomy/200583_out.krona.html]

Javascript must be enabled to view this page.

members
magnitude
magnitudeUnassigned
count
unassigned
taxon
rank

200583\_out

6

6
2
superkingdom

phylum
1224
6

1236
6
class

91347
6
order

6
543
family


SRS146888\_contig\_number\_contig-100\_723.96716
1
genus
6
561


SRS012273\_contig\_number\_45011SRS049402\_contig\_number\_contig-100\_160.116882SRS077127\_contig\_number\_contig-100\_14.42524SRS144362\_contig\_number\_28756SRS147022\_contig\_number\_10242
species
5
562
